# The effectiveness and safety of moxibustion for treating benign prostatic hyperplasia: A protocol for systematic review and meta-analysis

**DOI:** 10.1097/MD.0000000000022006

**Published:** 2020-09-04

**Authors:** Hui Chen, Qi Li, Ting Fang, Anting Liao, Shanshan Xiang, Zheng Guo, Mei Chen, Yongqiang Guo, Fushui Liu, Fengyun Yang

**Affiliations:** a Jiangxi University of Traditional Chinese Medicine; b Jiangxi Normal University; c The Affiliated Hospital of Jiangxi University of Traditional Chinese Medicine, Nanchang, China.

**Keywords:** benign prostatic hyperplasia, moxibustion, protocol, randomized controlled trial, systematic review and meta-analysis

## Abstract

**Background:**

Benign prostatic hyperplasia (BPH) is a disease of the urinary system. It is common in middle-aged and elderly men. Moxibustion is widely used to manage BPH and the associated lower urinary tract symptoms, but there is still lack of systematic review of moxibusiton for BPH. So the aim of this review is to comprehensively evaluate the effectiveness and safety of moxibustion in the treatment of BPH.

**Methods:**

The following 8 electronic databases including PubMed (1966–2020), EMbase (1980–2020), the Cochrane Library, Web of Science (1900–2020), China National Knowledge Infrastructure Database (1979–2020), WanFang Database (1998–2020), Chinese Scientific Journal Database (1989–2020), and Chinese Biomedical Literature Database (1979–2020) will be searched. No language restrictions will be used. Researchers will retrieve databases, identify trials, extract data, and evaluate the quality of eligible randomized controlled trials, independently. The outcomes will include: total effective rate, the American Urologic Association Symptom Score, International Prostate Symptom Score, urinary flow rate (measured in mL/s), changes in prostate size (measured in cc), quality of life, side effects and adverse events. The quality of methodology and evidence will be rated by using the Cochrane risk-of-bias assessment tool and grading of recommendations, assessment, development, and evaluation tool, respectively. Data synthesis will be presented by the manager of the Cochrane Collaboration's RevMan 5.3.0.

**Results:**

We will show the results of this study in a peer-reviewed journal.

**Conclusions:**

The findings will provide credible clinical evidence of moxibustion treatment for BPH.

**PROSPERO Registration number:**

CRD42020190630.

## 1 Introduction

Benign prostatic hyperplasia (BPH) is a disease of the urinary system. It is common in middle-aged and elderly men. Its global morbidity is high, and the patients have a younger trend. BPH patients often have the following lower urinary tract symptoms, such as nocturia, hesitancy, frequent urination, urgency, incontinence, and dysuria.^[1,2]^ Although it is not life-threatening, it has a high morbidity in the male elderly and seriously affects their quality of life.^[3]^ The prevalence of BPH rises markedly with increased age.^[4]^ Among men over the age of 50 years, 50% to 75% have experienced BPH.^[5]^ BPH is affected by a combination of factors such as age, family history, obesity, hypertension, diabetes, and poor lifestyle.^[6,7]^ Although there are various clinical treatments for BPH, no effective treatment has been found for all symptoms of BPH.^[8]^ All guidelines suggest a risk-adapted, individualized approach.^[9]^ Nowadays, the treatment methods for BPH are mainly conservative therapy.^[10,11]^ For mild to moderate male patients, it is suggested to select conservative treatments first such as watching-waiting, behavior, diet changing, and so on, and then pharmacologic treatment. However, this conventional treatment is limited by certain side effects. Such as adverse drug reactions. In order to avoid the impact of this limitation, some complementary and alternative medicine treatments have been applied clinically, but they exist mainly as health complementary therapies. It is suitable for BPH patients with mild clinical symptoms who are unwilling to receive standard therapies.^[12]^

Moxibustion, as a complementary and alternative therapy, has been proved effective in the treatment of urinary diseases.^[13]^ Moxibustion is a nonpenetrating therapy of acupuncture that uses heat from the burning of herbal preparations to stimulate specific acupoints in the body. Moxibustion has advantages of easy operation, low cost, nontoxic side effects, and clear curative effect. Clinically, acupuncture and moxibustion are often used to treat lower urinary tract symptoms in Asian countries.^[14]^ Based on the published studies on moxibustion for BPH, there is a lack of high-quality evidence recently. So the aim of this review is to comprehensively evaluate the effectiveness and safety of moxibustion in the treatment of BPH.

## 2 Methods

### 2.1 Study registration

This systematic review and meta-analysis protocol has been documented on PROSPERO 2020 CRD42020190630, a specialized systematic review registration platform. It could be obtained from: http://www.crd.york.ac.uk/PROSPERO/display_record.php?ID=CRD42020190630. We will conduct this protocol in line accordance with the statements of the Cochrane Handbook for Systematic Reviews and Meta-Analysis Protocol (PRISMA-P).^[15]^ If there are any changes in the protocol, we will describe it in our full manuscript.

### 2.2 Inclusion criteria

#### 2.2.1 Types of studies

All relevant randomized controlled trials (RCTs) and quasi-RCTs will be included.

#### 2.2.2 Types of participants

The patients who are diagnosed with BPH regardless of ethnic group, severity, syndrome type, and source of cases clinically will be included. Meanwhile, males and patients over the age of 18 are eligible. Studies that BPH combined with other basic diseases will be excluded. There are clear diagnostic criteria, the level of diagnosis is not limited. Diagnostic criteria for BPH includes clinical symptoms (nocturia, frequency, urgency, hesitancy, weak urinary stream, etc), digital rectal examination, urodynamic examination, imaging examination (prostate ultrasonography, color Doppler ultrasound of urinary system).

#### 2.2.3 Types of interventions/comparator(s)

The trial group will be treated with moxibustion or the combination therapy with other treatments (eg, western medicine, etc). Studies that moxibustion is used as a complementary treatment will be excluded. The control group will be treated with conventional standard treatments, such as western medicines, sham moxibustion, placebo or blank control, and so on.

#### 2.2.4 Types of outcome measures

##### 2.2.4.1 Primary outcomes

The main outcome indicators are listed below as follows:

(1) total effective rate;
(2) the American Urologic Association Symptom Score;
(3) International Prostate Symptom Score.

##### 2.2.4.2 Secondary outcomes

The secondary outcome indicators are listed below as follows:

(1) Urinary flow rate (measured in mL/s);
(2) changes in prostate size (measured in cc);
(3) Quality of life;
(4) Side effects and adverse events.

### 2.3 Exclusion criteria

The following exclusion criteria are presented:

(1) duplicate literature;
(2) Expert experience or case report;
(3) Theoretical or basic research;
(4) The diagnostic criteria and efficacy evaluation criteria are not clear;
(5) Result data is missing.

### 2.4 Search methods for identification of studies

#### 2.4.1 Electronic searches

The following 8 electronic databases including PubMed (1966–2020), EMbase (1980–2020), the Cochrane Library, Web of Science (1900–2020), China National Knowledge Infrastructure Database (1979–2020), WanFang Database (1998–2020), Chinese Scientific Journal Database (1989–2020), and Chinese Biomedical Literature Database (1979–2020) will be searched from the inception until September 2020. No language restrictions will be used. Searches will be re-run before the final analysis. The research will start following the PRISMA statement strictly. Only human subjects will be included. The main search terms include: “moxibustion,” “benign prostatic hyperplasia,” and “randomized controlled trial.” We will carry out literature retrieval in all electronic databases according to the pre-set literature retrieval strategy, which is based on Pubmed. As shown in Table 1.

**Table 1:**
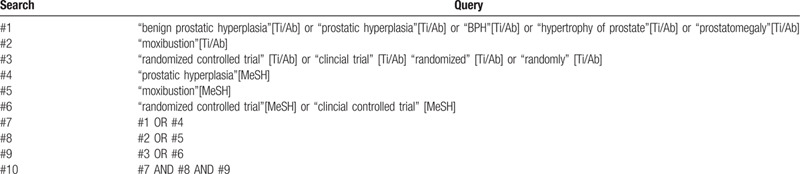
PubMed: will be searched on: September, 2020.

### 2.5 Studies selection

**Figure 1:**
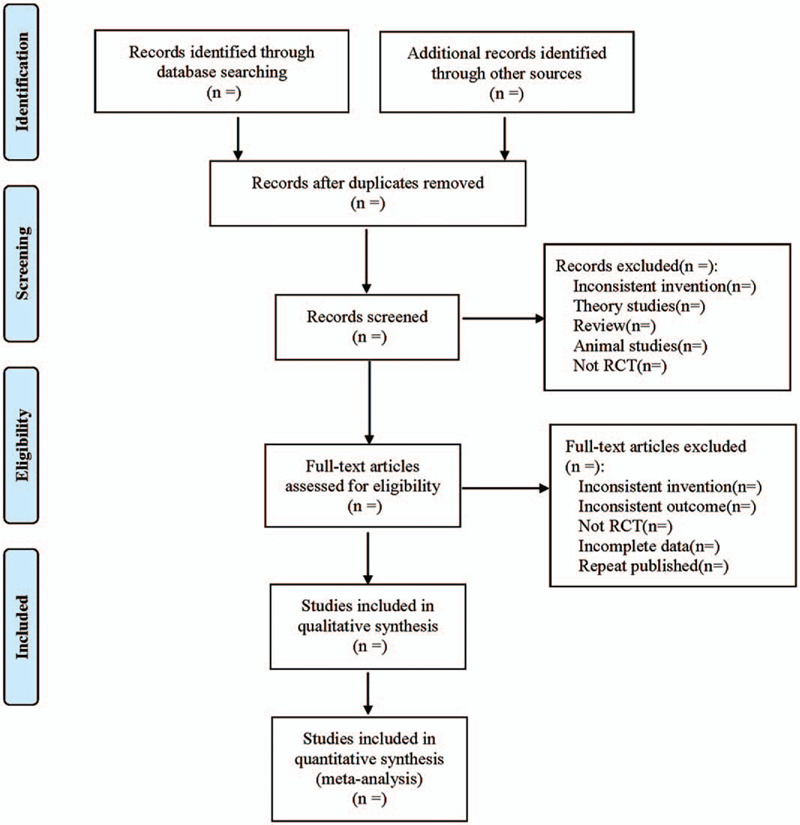
Flowchart of literature selection. Searching strategy.

All literature retrieved from the electronic database will be imported into the NoteExpress3.2.0 software for classification management, and duplicate checked out and published literature will be excluded. Before selecting the literature, all researchers will discuss and determine the selection criteria. According to the screening requirements of “rough screening first, then accurate screening,” first, 3 reviewers (Hui Chen, Zheng Guo, and Shanshan Xiang) will read the titles and abstracts back to back to eliminate literature that obviously did not meet the inclusion criteria. Then, the remaining literature and those literature that cannot be included after reading the title and abstract are further checked by downloading the full text to make a final decision. If there is any disagreement, it will be resolved by consensus or arbitrated by a fourth investigator (Fushui Liu). The filtering process is shown in Figure 1.

### 2.6 Data extraction and management

The 3 researchers (Hui Chen, Zheng Guo, and Shanshan Xiang) will extract the data independently from the pre-established data extraction table. The basic information extracted will be as follows: author, country, diagnostic criteria, age, sample size, intervention measures, control measures, course of treatment, outcome indicators, loss and follow-up, adverse reactions, random methods. Results will be cross-checked by 3 reviewers (Hui Chen, Zheng Guo, and Shanshan Xiang), respectively. If there is any disagreement, it will be resolved by consensus or arbitrated by a fourth investigator (Fushui Liu). Besides, the intention-to-treat analysis will be used to deal with the missing data. NoteExpress3.2.0 and Excel 2007 software will be applied to extract eligible data. When important data in the study is incomplete or missing, we will choose to contact the first author or corresponding author by phone or email to obtain the data.

### 2.7 Assessment of the methodological quality

Three reviewers (Hui Chen, Zheng Guo, and Shanshan Xiang) will follow the Cochrane Risk of Bias Assessment tool of Cochrane Reviewer's Handbook 5.0.24^[16]^ to evaluate the methodological quality of included RCTs back to back. The details are as follows: random sequence generation (selection bias), allocation concealment (selection bias), blinding of outcome assessment (detection bias), blinding of participants and personnel (performance bias), incomplete outcome data (attrition bias), selective reporting (reporting bias), and other bias. Results will be cross-checked by 3 reviewers (Hui Chen, Zheng Guo, and Shanshan Xiang), respectively. When important data in the study is incomplete or missing, we will choose to contact the first author or corresponding author by phone or email to obtain the data. If there is any disagreement, it will be resolved by consensus or arbitrated by a fourth investigator (Fushui Liu).

### 2.8 Assessing the level of evidence quality

Three evaluators (Hui Chen, Zheng Guo, and Shanshan Xiang) will independently select grading of recommendations, assessment, development, and evaluation tool to evaluate the level of evidence quality by using the software of GRADEprofiler 3.6. We will summarize the table information, as follows: Sample size, *I*^2^ value, *P*-value, risk of bias, inconsistency, inprecision, indirectness and publication bias, effect (95% confidence interval [CI]), and level of evidence. We will down the levels of the included RCTs from 5 aspects (risk of bias, inconsistency, inprecision, indirectness, and publication bias) to finally determine the quality of the evidence for each outcome indicator, expressed as high, medium, low, and extremely low.^[18]^

### 2.9 Data synthesis

#### 2.9.1 Measures of treatment effect

Review manager RevMan 5.3.0 software will be used for meta-analysis. The dichotomous variables will be summarized using risk ratio value with 95% CI, and the continuous variables will be summarized using mean difference/standardized mean difference with 95% CI. When the number of studies included for each outcome measure is less than 2, only descriptive analysis will be conducted to summarize the results.

#### 2.9.2 Heterogeneity analysis

We will strictly follow the criteria (*P* > .1 and *I*^2^ < 50%) to assess the statistical heterogeneity between included studies, and to show it in the form of forest plots. When *P* > .1 and *I*^2^ < 50%, the fixed effect model will be used for analysis for its lower heterogeneity; When *P* < .1 and *I*^2^ > 50%, the random effect model will be used for analysis for its higher heterogeneity. When the heterogeneity of included studies is evident, we will select subgroup analysis or sensitivity analysis to seek possible sources from clinical and methodological perspectives.

#### 2.9.3 Publication bias

The publication bias will be detected by using an funnel plots developed by Egger when the number of eligible RCTs ≧10.^[17]^

#### 2.9.4 Subgroup analysis

If the data are complete and available, we will perform subgroup analysis according to different control groups, age, course of treatment, study quality, and so on.

#### 2.9.5 Sensitivity analysis

For each outcome indicator with high heterogeneity, we will conduct sensitivity analysis from the aspects of sample size, quality, and age. Studies with high heterogeneity will be excluded to obtain stable results.

## 3 Discussion

BPH is a proliferative disease of glandular cells and stromal cells under the action of multiple factors (androgens, estrogens, growth factors, inflammation, etc).^[18]^ The prostate gland grows with men age, often causing some urinary tract symptoms, and even affecting patients’ mental health, which also urges patients to seek clinical treatment. With the accelerating aging of the population, BPH has become a health problem which has attracted much attention from the society. Therapeutically, western medicine therapy has been shown to be clinically effective in improving symptoms in patients with BPH.^[19]^ However, the medicines have significant side effects, such as dizziness, rhinitis and abnormal ejaculation,^[20]^ and so on. Particularly, if BPH is treated with a combination of 2 or more medicines, the adverse events are obvious; therefore, moderate to severe BPH patients should be used with caution.^[21]^

Moxibustion, a traditional Chinese medicine therapy, mainly involves the stimulation of specific acupoints by using heat, and it was often used in the treatment of BPH clinically.^[22]^ The warm and hot stimulation of moxibustion on the specific part of the human body, making the blood vessels dilate, enhance the hemodynamic, reduce the vascular resistance, thus effectively reducing the blood viscosity, and improve the microcirculation obstacle and hemorheology abnormality in BPH patients.^[23,24]^ Mechanism of moxibustion in the treatment of prostatic hyperplasia could be promoted the testosterone, estradiol and testosterone and estradiol proportion in the patients’ body, so as to make the increased androgen more bound to the human sex hormone binding protein, make the stromal cells are relatively stable, then maintain the size of the prostate volume, and moxibustion reduces the prostate-specific antigen(PSA) levels in the patients’ body, which regulates the relative stability of the prostate epithelial cells, and the corresponding adjustment of prostate volume.^[25]^ Considering that there are few reports on the mechanism of moxibustion in treating BPH, it is suggested that researchers strengthen the clinical research on moxibustion in treating BPH, so as to better serve the clinicians.

At present, there are many clinical trials to study the clinical effectiveness of moxibustion on BPH. However, there is a lack of systematic review and meta-analysis of moxibustion therapy for BPH. Thus, we intend to demonstrate the effectiveness and safety of moxibustion for BPH by conducting a high-quality systematic review and meta-analysis. In addition, this is the first protocol to comprehensively evaluate the clinical effectiveness of moxibustion in patients with BPH. We hope that the results of this review will provide clinicians with more reliable evidence-based evidence for treating BPH.

## Author contributions

**Conceptualization:** Hui Chen, Fushui Liu, Qi Li.

**Data curation:** Hui Chen, Zheng Guo, and Shanshan Xiang.

**Formal analysis:** Hui Chen, Zheng Guo, and Shanshan Xiang.

**Investigation:** Fushui Liu, Hui Chen.

**Methodology:** Ting Fang, Mei Chen, Yongqiang Guo.

**Software:** Anting Liao, Yongqiang Guo.

**Supervision:** Fushui Liu, Ting Fang, Fengyun Yang.

**Writing – original draft:** Hui Chen, Fushui Liu, Zheng Guo, and Shanshan Xiang.

**Writing – review & editing:** Fengyun Yang, Ting Fang, Mei Chen, Anting Liao, and Yongqiang Guo.
